# Expression of Calcitonin Gene-Related Peptide in Efferent Vestibular System and Vestibular Nucleus in Rats with Motion Sickness

**DOI:** 10.1371/journal.pone.0047308

**Published:** 2012-10-09

**Authors:** Wang Xiaocheng, Shi Zhaohui, Xue Junhui, Zhang Lei, Feng Lining, Zhang Zuoming

**Affiliations:** 1 Department of Clinical Aerospace Medicine, Key Laboratory of Aerospace Medicine of Ministry of Education, The Fourth Military Medical University, Xi'an, China; 2 Department of Otolaryngology Head and Neck Surgery, Xijing Hospital, The Fourth Military Medical University, Xi'an, China; Texas A&M University, United States of America

## Abstract

Motion sickness presents a challenge due to its high incidence and unknown pathogenesis although it is a known fact that a functioning vestibular system is essential for the perception of motion sickness. Recent studies show that the efferent vestibular neurons contain calcitonin gene-related peptide (CGRP). It is a possibility that the CGRP immunoreactivity (CGRPi) fibers of the efferent vestibular system modulate primary afferent input into the central nervous system; thus, making it likely that CGRP plays a key role in motion sickness. To elucidate the relationship between motion sickness and CGRP, the effects of CGRP on the vestibular efferent nucleus and the vestibular nucleus were investigated in rats with motion sickness.

**Methods:**

An animal model of motion sickness was created by subjecting rats to rotary stimulation for 30 minutes via a trapezoidal stimulation pattern. The number of CGRPi neurons in the vestibular efferent nucleus at the level of the facial nerve genu and the expression level of CGRPi in the vestibular nucleus of rats were measured. Using the ABC method of immunohistochemistry technique, measurements were taken before and after rotary stimulation. The effects of anisodamine on the expression of CGRP in the vestibular efferent nucleus and the vestibular nucleus of rats with motion sickness were also investigated.

**Results and Discussion:**

Both the number of CGRPi neurons in the vestibular efferent nucleus and expression level in the vestibular nucleus increased significantly in rats with motion sickness compared to that of controls. The increase of CGRP expression in rats subjected to rotary stimulation 3 times was greater than those having only one-time stimulation. Administration of anisodamine decreased the expression of CGRP within the vestibular efferent nucleus and the vestibular nucleus in rats subjected to rotary stimulation. In conclusion, CGRP possibly plays a role in motion sickness and its mechanism merits further investigation.

## Introduction

Motion sickness is a common and challenging problem. Various physiological measurements for this problem have been tested. However, no single parameter has yet been found to have a high enough sensitivity and specificity for the diagnosis or prediction of individual susceptibility to motion sickness. [1]–[3] Motion sickness may be precipitated by conflicting sensory input – visual and vestibular signals that do not match an internal model of expected environmental stimuli. [1]–[4] It is well known that a functioning vestibular system is essential for the perception of motion sickness. [5] Theoretically, if sufficiently provocative motion stimulus is introduced, anyone with a functioning vestibular system could be susceptible. [4] However, thus far the underlying mechanism is unclear. The innervations of the vestibular system include both the afferent and efferent vestibular system (EVS). Vestibular sensory organs in the inner ear are innervated by true efferent fibers originating from brainstem neurons. Studies show that electrical stimulation of EVS fibers can result in both facilitatory and inhibitory modulation of the sensory activity in the afferent vestibular system. [6] Therefore, EVS is considered to play a role in the modulation of the afferent input from the peripheral vestibular receptors to the central nervous system. [7]

Originally, efferent vestibular neurons (EVN) were assumed to be cholinergic, but currently, more evidence demonstrates that the efferent vestibular neurons contain both calcitonin gene-related peptide (CGRP) and choline acetyltransferase (CHAT). CGRP is a peptide with 37 amino acid residues translated from alternative processing of mRNA transcribed from the calcitonin gene. [8]–[10] CGRP is widely distributed in the central nervous system including the vestibular pathways. CGRP can be detected in the efferent pathways of the vestibular end-organs and the central vestibular system. [7], [11]–[13] Therefore, the role of CGRP in modulating this afferent input into the central nervous system is of fundamental importance in understanding neural processing in general and in the etiology of motion sickness. However, at present, much effort has been directed toward the understanding of the mechanism of CHAT in motion sickness, and anticholinergics are the most commonly used pharmacological agents today for prevention and treatment of this problem. [5] It is, therefore, necessary to explore the relationship between CGRP and the vestibular system and the role of CGRP in motion sickness.

We hypothesize that the EVS plays a role in the process of motion sickness via CGRP. To the best of our knowledge, there is little information about this. In this study, we establish an animal model of motion sickness in rats by rotary stimulation for 30 minutes in a trapezoidal stimulation pattern. We then measure and compare the number of CGRPi neurons in the vestibular efferent nucleus (VEN) of the brainstem at the level of the genu of the facial nerve and the level of expression of CGRP immunoreactivity in the vestibular nuclei of the brainstem. The measurements were taken before and after rotary stimulation by utilizing the immunohistochemistry technique. The effect of anisodamine (an anticholinergic) on the expression of CGRP within the vestibular efferent nucleus and the vestibular nucleus were also investigated. This study attempts to present a promising new direction in exploring the mechanism, prevention, and treatment of motion sickness.

## Materials and Methods

### Animals

All experiments used white, male Sprague-Dawley (SD) rats weighing about 220 g each. All animal procedures described in this study were performed in adherence with the Guide for the Care and Use of Laboratory Animals published by the US National Institutes of Health (NIH Publication No. 85–23, revised 1996) with approval from the Committee on the Ethics of Animal Experiments of the Fourth Military Medical University. All surgery was performed under diethyl ether anesthesia, and all efforts were made to minimize suffering.

### Establishment of an animal model of motion sickness

#### Rotary stimulation equipment and method

A mini-type animal centrifuge unit (Yongdao Medicine Instrument Company, Japan) was used to create an animal model of motion sickness. The unit is composed of a generator and an arm with two suspended plexiglass cages. The radius from the center of rotation to the point of suspension of the cages is 0.6 m. The angular acceleration, angular velocity, and run-time are controlled by computer. The cages not only revolve around a vertical axis but also can move along the direction of the arm during rotation. The rats can move around freely in the cages.

The cages were accelerated at 10^0^/s^2^ to a peak speed of 240°/s and rotated at peak speed for 5 minutes. Then the cages were decelerated at 10°/s^2^ to 0°/s. After that, the clockwise rotation alternated with the counterclockwise rotation. This stimulation lasted 30 minutes. Thus, the rats were treated with 1.46 G gravity force for 5 minutes – +10°/s^2^ angular acceleration, 10 s and −10°/s^2^ angular acceleration, 10 s tautologically, and 0.41/s^2^ Coriolis cumulative acceleration in each stimulation. [14]

#### Verification of an animal model of motion sickness by conditioned taste aversion

Conditioned taste aversion (CTA) tests were performed to verify that the animal model of motion sickness induced by the rotary stimulus was valid. Forty (40) rats were divided into 4 groups (10 per group) using a random digit table (Table 1). The 4 groups were: A) rotary stimulation undosed; B) rotary stimulation saline dosed; C) rotary stimulation anisodamine dosed; D) control group. Rats in groups A, B, and C were all subjected to rotary stimulation for 30 minutes. Thirty (30) minutes before rotary stimulation, group C was administered anisodamine orally (0.1 mg/100 g body weight) and group B was administered saline orally (0.1 mg/100 g body weight). [5] The control group had no rotary stimulation.

**Table 1:** Experimental groups for verifying the animal model of motion sickness.

group	rotary stimulation	saline dosed	anisodamine dosed
A	30 minutes	−	−
B	30 minutes	+	−
C	30 minutes	−	+
D	No	−	−

After rotary stimulation, all 4 groups were given water with 0.15% saccharin. The intake volume of saccharin solution was measured every 24 h. The volumes consumed in the first 24 h, second 24 h, and third 24 h after stimulation were compared with the volume in the 24 h prior to the stimulation. [15]

### Immunohistochemistry

Fifty (50) rats were divided into 5 groups (10 per group) using a random digit table (Table 2). The 5 groups were: I) triple rotary stimulation undosed; II) single rotary stimulation undosed; III) single rotary stimulation saline dosed; IV) single rotary stimulation anisodamine dosed; V) control group. Rats in group I were subjected to rotary stimulation 3 times at 24 h intervals, and groups II, III, and IV were subjected to rotary stimulation once. The control group was not subjected to rotary stimulation. Thirty (30) minutes before rotary stimulation, rats in group IV were administered anisodamine (0.1 mg/100 g body weight) orally and rats in group III were administrated saline (0.1 mg/100 g body weight).

**Table 2:** The experimental groups for immunohistochemistry.

group	rotary stimulation	saline dosed	anisodamine dosed
I	3 times	−	−
II	once	−	−
III	once	+	−
IV	once	−	+
V	No	−	−

Rats were anesthetized with diethyl ether after undergoing single or triple rotary stimulation. Cardiac perfusions were performed with phosphate buffered saline (200 ml, pH 7.2, 5 min) followed by 4% paraformaldehyde in 0.1 M phosphate buffer (500 ml, pH 7.4, 20 min). The brain was then removed and fixed in 4% paraformaldehyde in 0.1 M phosphate buffer (pH 7.4) for 4 h before being stored overnight (4°C) in 0.1 M phosphate buffer (pH 7.4) containing 30% sucrose. The next day, transverse serial sections (30 µm) were sliced through the brainstem from the hypoglossal nucleus to the anterior aspect of the parabrachial nucleus on a sliding microtome (CM1900 cryostat manufactured by Leica, Germany) and collected in 0.1 M phosphate buffer (pH 7.4, 4°C). Alternate sections were collected and divided into 2 groups. The tissue sections from the first group were immunohistochemically labeled for CGRP, and 10–20 sections were incubated in plastic boats with rabbit anti-CGRP (diluted 1∶1,000, Chemico Company, Ltd, Malaysia) overnight at 4°C. Antibody-antigen binding was visualized using an avidin-biotin-peroxidase complex (ABC Kit, Vector Laboratories, America) with 3, 3″-diaminobenzidine tetrahydrochloride (Sigma-Aldrich Chemicals, America) as the chromogen. After the reaction, brainstem sections were mounted on chrome alum-gelatin-coated microscope slides, air-dried, dehydrated in ethanol, cleared in xylene, and cover-slipped with Permount. [13], [16]–[18]

To ensure specificity of the CGRP staining procedure, the tissue sections from the second group were incubated in goat blood serum instead of rabbit anti-CGRP. The visualization steps were the same as for the first group. No immunoreactive signal was observed in these sections, indicating that any observed immunoreactivity was due to CGRP and not non-specific binding.

All tissue sections were examined with an Olympus Vanox-T microscope using bright field illumination. The vestibular efferent nucleus, as described by Tanaka et al. and Wackym et al., and the vestibular nucleus were studied. [18], [19] The cell bodies of CGRP positive and CGRPi fibers were identified and photographed.

### Densitometry

The dorsolateral to the genu of the facial nerve (DL), the medial to the genu of facial nerve (M), and the caudal pontine reticular nucleus (CPR) regions were examined for CGRP labeled cells. After initial evaluation, labeled cells in DL, M, and CPR regions were counted using a Leica Q-500 microscope fitted with image analysis hardware and software for densitometry and particle analysis (Microcomp Image Analysis System, Southern Micro Instruments, Inc., Atlanta GA). Then, the average background gray levels were determined for each stained batch of sections. Pixels with gray levels at least 10% darker than the average background levels were defined as positive for CGRP labeling. This resulted in a two-level image (black/white) that was further processed to identify contiguous pixels so that neuronal cell bodies could be automatically recognized and counted. [17] Gray scale of CGRP fibers in the vestibular nucleus was also quantitatively analyzed. Five (5) HP visual fields in the vestibular nucleus were selected to evaluate the optical density of CGRP fibers in each section. The optical density of CGRP immunohistochemistry in the vestibular nucleus was calculated as the optical density observed minus the background density.

### Image statistical analysis

Data is expressed as a mean±S.D. All statistical analyses were done using the SPSS 8.0 statistical software package. The intake volumes of 0.15% saccharin solution before and after stimulation within each group were statistically analyzed with univariate ANOVA. The reductions in intake volumes after stimulation were calculated as the volume after stimulation minus the volume before stimulation in the 4 groups and the differences between the groups were also compared with univariate ANOVA. P values<0.05 were deemed significant.

Five (5) tissue sections were selected from each rat after immunohistochemistry. The number of CGRP positive cells and optical density were statistically analyzed with univariate ANOVA. Statistical significance of the differences between groups was evaluated using the Student-Newman-Keuls test. [17] Significance was assigned at the *P*<0.05 level.

## Results

### Rotary stimulation and conditioned taste aversion

**Figure 1:**
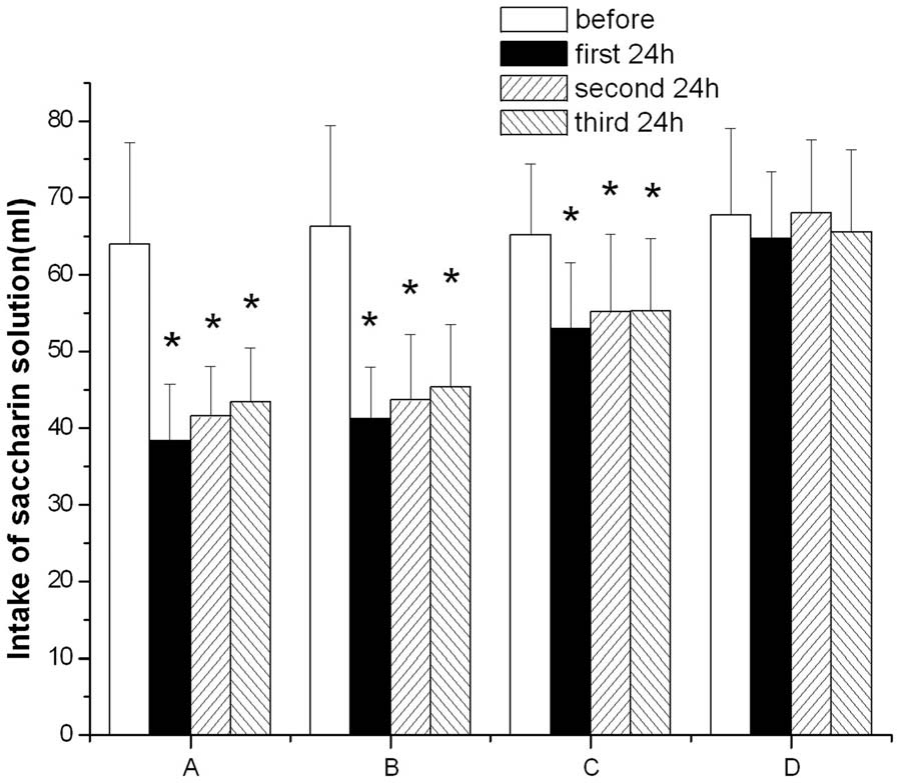
Comparison of intake volume of saccharin solution before and after rotary stimulation in the four experimental groups. A: rotary stimulation undosed; B: rotary stimulation saline dosed; C: rotary stimulation anisodamine dosed; D: control group. *P<0.05 as compared with the before rotary stimulation respectively. The reduction in group C was significantly less than those of group A and group B in the three 24 h intervals after rotary stimulation.

The average intake volume of saccharin solution was 64.0 ml before rotary stimulation in group A, while the average intake volumes were 38.4 ml, 41.6 ml, and 43.3 ml in the first 24 h, second 24 h, and third 24 h after stimulation, respectively (Table 3). In group A, the intake volumes in the first 24 h, second 24 h, and third 24 h after stimulation were 60.0%, 64.9%, and 67.8%, compared with that before stimulation. Results of such comparisons were 62.1%, 65.8%, and 68.4% in group B; and 81.1%, 84.6%, and 84.8% in group C, respectively. Saccharin solution intake after the rotary stimulation was reduced in groups A, B and C compared to that prior to the stimulation (Figure 1). The differences were significant in the 3 groups (P<0.05). Although the intake volumes were reduced in the 3 groups after stimulation, the reduction in group C was significantly less than those of group A and group B in the three 24 h intervals. The reductions show no significant difference between group A and group B in the 3 24 h intervals. Animals from group D showed no significant intake volume change during the experimental period.

**Table 3:** Comparison of reduction in intake volumes (%) in the 4 experimental groups.

group	1st 24 h	2nd 24 h	3rd 24 h
**A**	60.0	64.9	**67.8**
**B**	62.1	65.8	68.4
**C**	81.1*	84.6*	84.8*
**D**	95.4	100.4	96.8

A rotary stimulation undosed;B rotary stimulation saline dosed; C rotary stimulation anisodamine dosed;D control group. *P<0.05 as compared with groups A and B, respectively.

### Expression of CGRP in the vestibular efferent nucleus and the vestibular nucleus

**Figure 2:**
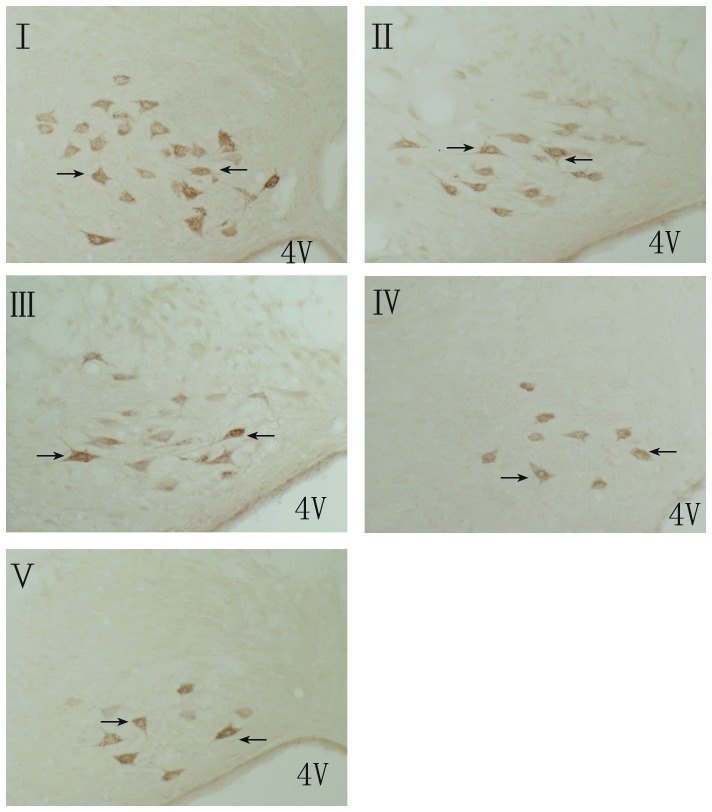
The CGRP positive cells in the vestibular efferent nucleus(magnification:10×20). I: triple rotary stimulation with undosed; II: single rotary stimulation with undosed; III: single rotary stimulation with saline dosed; IV: single rotary stimulation with anisodamine dosed; V: control group, 4 V: 4th ventricle. Arrows show the CGRP positive cells in the vestibular efferent nucleus.

**Figure 3:**
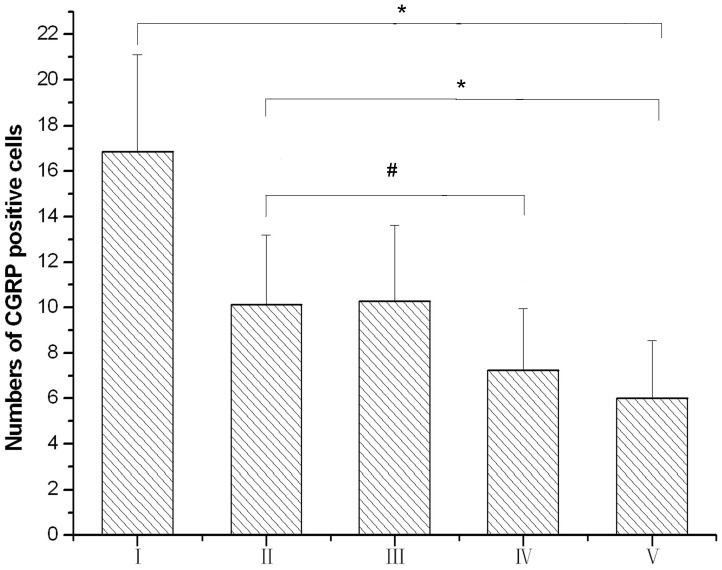
The numbers of CGRP positive cells in the efferent nucleus. I: triple rotary stimulation with undoesd; II: single rotary stimulation with undoesd; III: single rotary stimulation with saline dosed; IV: single rotary stimulation with anisodamine dosed; V: control group.*P<0.05 for group I vs. group V and group II vs. group V; #*P*<0.05 for group IV vs. group II.

The CGRP-positive EVN were located in the brainstem and were small fusiform-shaped neurons mainly composed of 3 groups of neurons: neurons dorsolateral to the genu of the facial nerve (DL); neurons dorsomedial to the genu of facial nerve (M); and scattered cells throughout the caudal pontine reticular nucleus (CPR). The average number of CGRPi neurons in the vestibular efferent nucleus of the brainstem were 16.86, 10.13, 10.28, 7.25, and 6.00 in groups I, II, III, IV, and V, respectively. The number of CGRPi neurons in the vestibular efferent nucleus increased significantly in groups I and II compared with group V (*P*<0.05). The increase of CGRPi neurons in group I was significantly greater than that in group II. The number of CGRPi neurons in the vestibular efferent nucleus was significantly decreased in group IV compared with that in groups I, II, and, III (*P*<0.05). There was no significant difference between groups II and III (Figures 2, 3).

**Figure 4:**
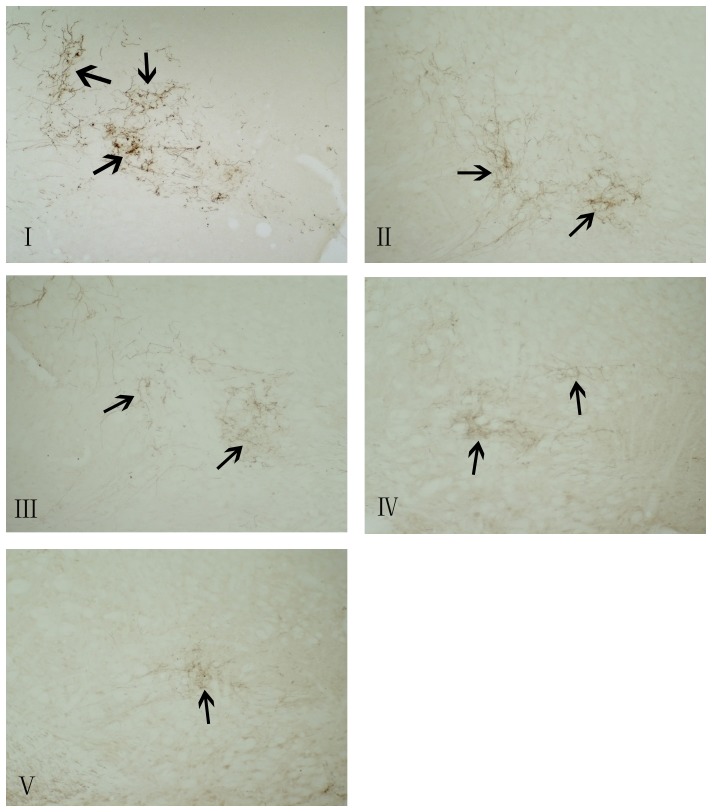
Expression of CGRPi fibers in the vestibular nuclei (magnification:10×10). I; triple rotary stimulation with undoesd; II: single rotary stimulation with undoesd; III:single rotary stimulation with saline dosed; IV:single rotary stimulation with anisodamine dosed; V: control group. Arrows show the CGRPi fibers in the vestibular nuclei.

**Figure 5:**
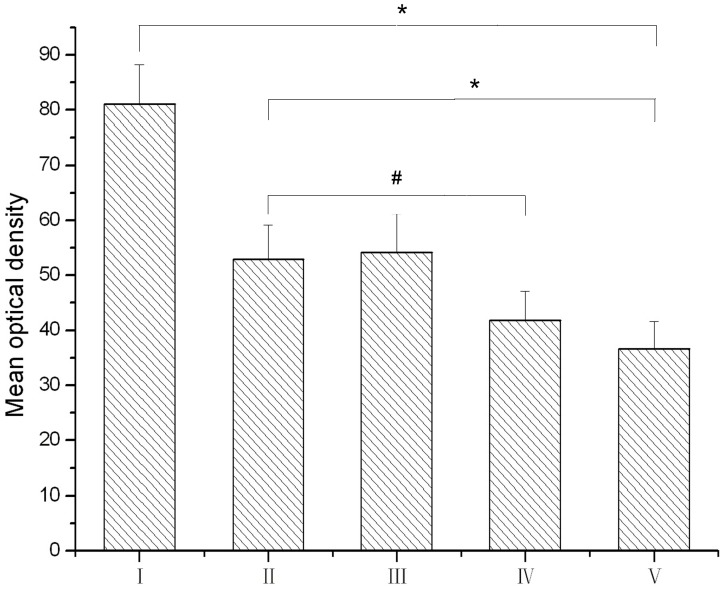
The mean optical density of CGRP immunoreactivity in the vestibular nuclei of rats. I: triple rotary stimulation with undoesd; II: single rotary stimulation with undoesd; III: single rotary stimulation with saline dosed; IV: single rotary stimulation with anisodamine dosed; V: control group. *P<0.05 for group I vs. group V and group II vs. group V; #*P*<0.05 for group IV vs. group II.

CGRPi fibers were observed in the vestibular nucleus (Figures 4, 5). The average optical density of CGRP immunohistochemistry in the vestibular nucleus were 81.1, 52.9, 54.2, 41.8, and 36.7 in groups I, II, III, IV, and V, respectively. The level of immunoreactivity in the vestibular nucleus increased significantly in groups I and II compared to group V (P<0.05). The increase in group I was greater than that in group II. In group IV, the level of such immunoreactivity in the vestibular nucleus was significantly decreased compared to groups I, II, and III. There was no significant difference between groups II and III.

## Discussion and Conclusion

Prevention and treatment of motion sickness is a challenge, particularly in aerospace medicine, due to its high incidence and unclear pathogenesis. At present, animal models of motion sickness have been developed in cats, dogs, rats, and squirrel monkeys. CTA and pica are classic indices to evaluate the animal model of motion sickness. CTA, a significant decrease in the animals' consumption of some substance with a certain taste (e.g., saccharin solution), can be induced by various stimuli. Pica, an increase in the animals' consumption of kaolin or other substances of no nutritional value, can also be induced. Today, CTA is extensively used to evaluate the animal model of motion sickness by observing the decrease in consumption of saccharin solution after stimulation, while pica is used by observing the increase in consumption of kaolin after stimulation. Recent studies suggest other new indices for the study of motion sickness by observing symptoms after rotation, such as piloerection, tremble, urinal and fecal incontinence. However, the utility and broad acceptance of these newer indices may require further investigation. [20] Compared to observing symptoms and Pica, CTA is sensitive, simple, stable, easy to perform, and applicable to a variety of animals. Furthermore, CTA is readily quantifiable and, consequently, more commonly used. Moreover, CTA is a behavioral index that, through the degree of antidipsia, reflects the severity of motion sickness. [14], [21] Therefore, in our experiments, we observe the changes in the intake volumes of saccharin solution before and after rotation to evaluate the animal model of motion sickness in rats. The intake volume of 0.15% saccharin solution was significantly reduced after motion stimulation. Antidipsia can be decreased by administration of anisodamine – an anticholinergic drug currently used to prevent and treat motion sickness. Observation of this effect further indicates the validity of our animal model using rotary stimulation for 30 minutes in a trapezoidal pattern.

The efferent vestibular neurons (EVN) are located in the vestibular efferent nucleus (VEN) and are mainly composed of 3 types of neurons – DL, M, and CPR. Many of the EVN are CGRPi and send efferent fibers to the vestibular end-organs. [13], [22], [23] In our experiments, the number of CGRPi neurons in VEN increased significantly in rats after rotary stimulation. Moreover, the increase of CGRPi neurons in rats after rotary stimulus was 3 times greater than that of the rats that underwent rotary stimulus only once.

The exact role of CGRP in EVS still is unclear today, but it has been found that CGRP increases the discharge firing rate of afferent fibers innervating the hair cells in the lateral line organ of Xenopus laevis. [19] In end-organs of the human vestibule, CGRPi is located in vesiculated nerve fibers and bouton-type nerve terminals that directly contact afferent nerve chalices surrounding type I sensory cells and afferent nerve fibers to form an *en passant* contact with afferent dendrites. [16], [22] It follows that the release of CGRP is able to directly alter primary afferent inputs via type I hair cells of the central vestibular nervous system. On the other hand, EVN contain both CGRP and choline acetyltransferase; and studies have found an interaction between CGRP and acetylcholine. [16], [22], [23] The acetylcholine-mediated efferent system is thought to provide a tonic inhibitory influence on the afferent activity arising from each vestibular receptor. Although such an interaction is not clear in the vestibular system, it has been reported that CGRP increases intracellular Ca^2+^ concentration in response to Ach in cochlear hair cells. [16] CGRP has also been shown to influence the expression of nicotinic acetylcholine receptor (nAChR) subunits in skeletal muscle which leads to an increase in cAMP levels and acetylcholine receptor synthesis. CGRP may have a similar regulatory role in the vestibular system. [13], [18] CGRP up-regulates synthesis of acetylcholine receptors and alters the sensitivity of primary afferent neurons to acetylcholine.

Recently, researchers report that the vestibular nuclei contain numerous afferent neurons that send projections to the vestibular efferent nucleus, some of which are CGRP cells. [24] This afferent innervation provides morphological evidence that EVN receive input from including CGRP cells. [24] These vestibular primary CGRP afferent neurons may have an influence on EVN. CGRP acts as an important co-transmitter and modulator in the afferent-mediated activity of vestibular efferent neurons, which in turn affect afferents in the vestibular end-organs. [13], [18], [24] In our study, we observe that the level of CGRP fiber immunoreactivity in the vestibular nucleus increases significantly in an animal model of motion sickness, and the increase after 3 rotary stimulation events is greater than after rotary stimulation once. It is unclear today whether the CGRP fibers are afferent to the vestibular efferent nucleus from CGRP afferent cells in the vestibular nucleus or efferent from VEN to the vestibular nucleus. However, the results of our current studies suggest that CGRP may have a potential role in motion sickness.

In our experiments, the number of CGRPi neurons in VEN and the level of CGRP fiber immunoreactivity in the vestibular nucleus increased significantly in rats following rotary stimulation. Our findings have provided further evidence to indicate an important role of CGRP in the pathogenesis of motion sickness and support additional neurophysiological and molecular biological studies.

Anisodamine and scopolamine are anti-cholinergic drugs and both are used to prevent and treat motion sickness. [25], [26] It has been proved that anisodamine and scopolamine have equal anti-motion sickness effect. Moreover, anisodamine does not induce drowsiness, blurred vision, or other side effects, as does scopolamine. Therefore, according to study of these two agents, anisodamine is more suitable for anti-motion sickness than scopolamine. [26] Accordingly, in our experiment, we selected anisodamine as an anti-motion sickness drug and investigated the effect of anisodamine on motion sickness and CGRP. Our results show that anisodamine administered orally before stimulation can alleviate the symptoms (i.e. CTA) of motion sickness (Figure1). Importantly, this study demonstrates for the first time that this medication can also significantly reduce the expression of CGRP in the vestibular efferent nucleus and the vestibular nucleus of rats that have undergone rotary stimulation (Figures 2, 5). This finding could be very helpful not only to further support a link between CGRP and motion sickness but also to motivate the use of CGRP as a potential biomarker for developing a specific test or new medication for motion sickness.

## References

[1] Shupak A, Gordon CR (2006) Motion sickness: advances in pathogenesis, prediction, prevention, and treatment. Aviat Space Environ Med 77: 1213–23. 17183916

[2] Buyuklu F, Tarhan E, Ozluoglu L (2009) Vestibular functions in motion sickness susceptible individuals. Eur Arch Otorhinolaryngol 266: 1365–1371. 19242710 10.1007/s00405-009-0927-6

[3] Kennedy RS, Drexler J, Kennedy RC (2010) Research in visually induced motion sickness. Appl Ergon 41: 494–503. 20170902 10.1016/j.apergo.2009.11.006

[4] Golding JF (2006) Motion sickness susceptibility. Auton Neurosci 129: 67–76. 16931173 10.1016/j.autneu.2006.07.019

[5] Zajonc TP, Roland PS (2006) Vertigo and motion sickness. Part II: Pharmacologic treatment. Ear Nose Throat J 85: 25–35. 16509240

[6] Chi FL, Wang ZM, Li KY, Wu W (1999) Calcitonin gene-related peptide changes in efferent vestibular system during vestibular compensation. Chinese Journal of Otorhinolaryngology 34: 11–13. 12764785

[7] Schrott-Fischer A, Kammen-Jolly K, Scholtz A, Rask-Andersen H, Glueckert R, et al (2007) Efferent neurotransmitters in the human cochlea and vestibule. Acta Otolaryngol 127: 13–9. 17364323 10.1080/00016480600652123

[8] Vause CV, Durham PL (2010) Calcitonin gene-related peptide differentially regulates gene and protein expression in trigeminal glia cells: Findings from array analysis. Neurosci Lett 473: 163–7. 20138125 10.1016/j.neulet.2010.01.074 PMC3131086

[9] Mapp PI, McWilliams DF, Turley MJ, Hargin E, Walsh DA (2012) A role for the sensory neuropeptide calcitonin gene-related peptide in endothelial cell proliferation in vivo. Br J Pharmacol 10: 1476–5381. 10.1111/j.1476-5381.2012.01848.x PMC3417445 22233274

[10] Huang J, Stohl LL, Zhou X, Ding W, Granstein RD (2011) Calcitonin gene-related peptide inhibits chemokine production by human dermal microvascular endothelial cells. Brain Behav Immun 25 787–99. 21334428 10.1016/j.bbi.2011.02.007 PMC3081395

[11] Kong WJ, Scholtz AW, Hussl B, Kammen-Jolly K, Schrott-Fischer A (2002) Localization of efferent neurotransmitters in the inner ear of the homozygous Bronx waltzer mutant mouse. Hear Res 167: 136–55. 12117537 10.1016/s0378-5955(02)00382-9

[12] Kong WJ, Scholtz AW, Kammen-Jolly K, Glückert R, Hussl B, et al (2002) Ultrastructural evaluation of calcitonin gene-related peptide immunoreactivity in the human cochlea and vestibular endorgans. Eur J Neurosci 15: 487–97. 11876776 10.1046/j.0953-816x.2001.01880.x

[13] Wackym PA, Popper P, Ward PH, Micewck PE (1991) Cell and molecular anatomy of nicotinic acetylcholine receptor subunits and calcitonin gene-related peptide in the rat vestibalar system. Otolaryngology-head and Neck surgery 105: 493–510. 1762788 10.1177/019459989110500401

[14] Fu J, Sun Z, Yu L, Liu L (2003) Comparison of Four Stimulation Patterns Inducing Motion Sickness. Journal of Jilin University (Medicine Edition) 29: 35–37.

[15] Jiang Z, Shen H, Yang K, Xu B, Zhou WR (2000) Establishment of conditioned taste aversion as a motion sickness model in rat. Chin J Naut Med 7: 97–100.

[16] Ohno K, Takeda N, Tanaka-Tsuji M, Matsunaga T (1993) Calcitonin gene related peptide in the efferent system of inner ear. A review. Acta Otolaryngol Suppl. 501: 16–20. 10.3109/00016489309126206 8447220

[17] Thompson GC, Ross CD, Thompson AM, Byers JM (1995) Changes in brainstem calcitonin gene-related peptide after VIIth and VIIIth cranial nerve lesions in guinea pig. Brain Res 683: 140–148. 7552338 10.1016/0006-8993(95)00364-v

[18] Wackym PA, Popper P, Micevych PE (1993) Distribution of calcitonin gene-related peptide mRNA and immunoreactivity in the rat central and peripheral vestibular system. Acta Otolaryngol (stockh) 113: 601–608. 8266786 10.3109/00016489309135871

[19] Tanaka M, Takeda N, Senba E, Tohyama M, Kubo T, et al (1989) Localization, origin and fine structure of calcitonin gene-related peptide-containing fibers in the vestibular end-organs of the rat. Brain Res 504: 31–35. 2598015 10.1016/0006-8993(89)91593-x

[20] Yu XH, Cai GJ, Liu AJ, Chu ZX, Su DF (2007) A novel animal model for motion sickness and its first application in rodents. Physiol Behav 92: 702–7. 17612582 10.1016/j.physbeh.2007.05.067

[21] Hu S, Willoughby LM, Lagomarsino JJ, Gaeger HA (1996) Optokinetic rotation-induced taste aversions correlate with over-all symptoms of motion sickness in humans. Percept Mot Skills 82: 859–864. 8774022 10.2466/pms.1996.82.3.859

[22] Ohno K, Takeda N, Yamano M, Matsunaga T, Tohyama M (1991) Coexistence of acetycholine and calcitonin gene-related peptide in the vestibular efferent neurons in the rat. Brain Res 566: 103–107. 1814528 10.1016/0006-8993(91)91686-u

[23] Perachio AA, Kevetter GA (1989) Coexistence of choline acetyltransferase and calcitonin gene-related peptide in vestibular efferents of the gerbil. Neurosci Abs15: 518.

[24] Chi FL, Jiao Y, Liu HJ, Wang J, Shi Y, Barr JJ (2007) Retrograde neuron tracing with microspheres reveals projection of CGRP-immunolabeled vestibular afferent neurons to the vestibular efferent nucleus in the brainstem of rats. Neuroendocrinology 85(3): 131–8. 17457027 10.1159/000101959

[25] Spinks AB, Wasiak J, Villanueva EV, Bernath V (2007) S copolamine (hyoscine) for preventing and treating motion sickness. Cochrane Database Syst Rev: CD002851. 10.1002/14651858.CD002851.pub3 17636710

[26] Jiang ZL, Shen HM, Yang K, Jin SY (1999) Observation of the effect of ginger and Ach-receptor inhibitors on simulated motion sickness in rats. Chin J Naut Med6: 20–22.

